# Physician and Patient

**Published:** 1863-05

**Authors:** 


### PHYSICIAN AND PATIENT.

A POOR young clerk, in a hardware store in New-York, had a heavy box fall on him in such a way as to peel the skin from the whole surface of the knee, and let out the sinovial fluid, that is, the liquid which nature prepares for lubricating the joint. Medical advice was taken, and but one opinion was given, that loss of life or limb, if not both, was inevitable. The youth had a widowed mother, who was dependent for a living on his scanty earnings; hence to both the mishap was a crushing calamity. While halting to decide whether to have the limb taken off at once, or to run the risk of losing life, in the hope that some favorable change might take place by which life might be saved, and the limb too, although hopelessly stiffened, an adventurous young surgeon told the youth that he thought his limb might be saved, and that if he made the trial and was successful, he could pay him at some future day, when he was more able. The boy expressed his gratitude in a gush of tears, through quivering lips. By this time the whole surface of the knee, covering many square inches, was an ugly sore, a mass of matter, and the surgeon's instrument could easily pass from one side of the limb to the other, under the knee-pan. The limb was firmly bound, the wound was washed, the ragged edges of the skin were trimmed off, nitrate of silver was injected into the joint, an incision was made for six or eight inches above and below the knee, and the skin was detached on either side of this line, until it was almost severed from the body, remaining attached by a few inches on the under side; this loose skin was then drawn over the knee, and so stretched that the edges met. In due time the youth got well, and saved his limb, so that it would not be perceived by his gait that any injury had been received. Years passed away; the kind-hearted surgeon became an old man, the boy a millionaire, and a most unprincipled dog besides, for he had not paid the doctor a dollar. "Well, my son, you are rich now; you own houses and lands and stocks to a large amount, and the doctor who saved your limb, if not your life, has never been paid. Shall I take him some money?" "Yes, mother, take him a hundred dollars." The pitiful scamp!

The most eminent surgeon in America was once called on to perform the operation for a club-foot, for a gentleman who had come to New-York from a long distance for the express purpose of securing the best talent in that line which money could procure. The operation was performed and resulted most happily; it was a beautiful success. The patient himself was a man of culture; the fascination of his manners was such, that the surgeon spent many hours in his company during the long weeks of treatment. The cure was complete, and a day appointed for the patient's departure. Upon the announcement that the fee was three hundred dollars, he appeared almost hurt that it should have been so trifling a sum, and requested that the surgeon should make it larger and present it for payment the next day, which would be the last visit. The next day the patient was missing, and was never heard of afterwards.

There is no class of men living who give more time and personal attention for the benefit of others, without any compensation, and without any hope of compensation, than educated medical men; it is a part of their profession; it is an engagement which they tacitly undertake, on the eventful occasion of receiving their diplomas, never to refuse a call of rich or poor, day or night, whether in town or country, unless in case of inability from actual sickness in their own persons. And how well the profession keep these implied promises, and even go beyond them in waiting on the sick and dying when they themselves ought to have been in bed, there are literally multitudes to testify.

There are some physicians who never make a charge, who never present a bill, but leave to their patients to pay when and what they please. There is no merit in this; it is a positive injury to any ordinary community; its operation is demoralizing, and it ought not to be countenanced. Such a course may be proper enough when the millennium comes, not before.

Some long-headed doctor has said that the best, the most willing paymasters for medical services, are rich heirs and legatees. That Nestor among surgeons, the kind-hearted Dr. Mott, who, by the way, is the youngest-looking old man in the nation, upon whose placid features none can look without a feeling of envy and of love, was accustomed to say to his classes many years ago: "Young gentlemen, in entering on the duties of your profession, have two pockets made, one very large, the other quite small; the former for the insults, the latter for the fees." In our own early experience, a very rich man refused to pay his bill. It was such a mortification to us, we resolved that thereafter, when we did "trust" a man, to let him pay it "on his own motion," otherwise let it go to the account of profit and loss. Two years later a letter came with a check for more than we ever asked for, because the man got sick again, and had faith in us.

We once prescribed for a young lady; it was a very tedious case, but she eventually regained her health, became the principal of a flourishing school, and made money largely. Seven years from our first acquaintance, she wrote for her bill, and on remitting it, with a bonus, she desired advice in reference to the result of an accident which threatened to destroy life eventually. But physicians of large and long experience know very well that these are exceptional cases, that three times out of four the relations of physician and patient are of a delightful character; there is trustingness, respect, and gratitude all combined, increasing with increasing years. It is to the family physician that secrets are communicated which are not to be told to parent or child, to husband or wife; secrets which would blast the reputation of whole families, which would put into commotion an entire community, and breed life-long enmities between the hitherto loved and loving. But who ever heard of these confidences being violated, of these holy trusts being forfeited? Rare indeed are the cases where domestic concord has been broken up by the dereliction of the medical attendant; and the fondness with which the name of the old family physician is mentioned, after he himself has passed away, by those who have sought his advice and have been benefited by his ministrations, is only second in a great many cases to that which is felt for father and mother, after they have gone to their long home.

Many are the cases where persons have felt themselves to be under obligations for deliverance from sufferings or impending death, or life-long deformities, which no money could ever cancel, and an affectionate gratitude springs up, which there is a sweetness in beholding, and is highly creditable to human nature. Hence it is that in cities and large towns it is of frequent occurrence that the same physician "practices" in families to the third generation, and his mantle falls upon his son.

